# Virtual Dissection: Emerging as the Gold Standard of Analyzing Living Heart Anatomy

**DOI:** 10.3390/jcdd7030030

**Published:** 2020-08-12

**Authors:** Justin T. Tretter, Saurabh Kumar Gupta, Yu Izawa, Tatsuya Nishii, Shumpei Mori

**Affiliations:** 1 Heart Institute, Cincinnati Children’s Hospital Medical Center, Cincinnati, OH 45229, USA; justin.tretter@cchmc.org; 2 Department of Pediatrics, University of Cincinnati College of Medicine, Cincinnati, OH 45229, USA; 3 Department of Cardiology, All India Institute of Medical Sciences, New Delhi 110029, India; drsaurabhmd@gmail.com; 4 Division of Cardiovascular Medicine, Department of Internal Medicine, Kobe University Graduate School of Medicine, Kobe, Hyogo 650-0017, Japan; u.izawa@gmail.com; 5 Department of Radiology, National Cerebral and Cardiovascular Center, Suita, Osaka 564-8565, Japan; ttsynishii@ncvc.go.jp; 6 UCLA Cardiac Arrhythmia Center, UCLA Health System, David Geffen School of Medicine at UCLA, Los Angeles, CA 90095, USA

**Keywords:** cardiac anatomy, computed tomography, congenital heart disease, three-dimensional imaging: virtual dissection, volume rendering

## Abstract

Traditionally, gross cardiac anatomy has been described mainly based on the findings in the dissection suite. Analyses of heart specimens have contributed immensely towards building a fundamental knowledge of cardiac anatomy. However, there are limitations in analyzing the autopsied heart removed from the thorax. Three-dimensional imaging allows visualization of the blood-filled heart in vivo in attitudinally appropriate fashion. This is of paramount importance for not only demonstration of cardiac anatomy for educational purposes, but also for the detailed anatomical evaluation in patients with acquired and congenital heart disease. In this review, we discuss the advantages of three-dimensional imaging, specifically focusing on virtual dissection, a volume rendering-based reconstruction technique using computed tomographic data. We highlight examples of three-dimensional imaging in both education and guiding patient management.

## 1. Introduction

The modern understanding of cardiac anatomy is incomplete without referencing the works of Professor Robert H. Anderson. He continues to be a pillar to which our knowledge of cardiac anatomy in both normal as well as malformed hearts is supported. During his long illustrious career, he has enriched the field of cardiac development and anatomy, the latter of which has largely depended on analysis of heart specimens. The autopsy-based analyses, however, have inherent disadvantages of loss of in vivo blood-filled shape and attitudinally appropriate relationship to surrounding structures. Besides, delineation of cardiac anatomy mandates dissection, which results in deformation and permanent loss of its original state.

Modern computed tomography scanners acquire high-resolution datasets. Simultaneous improvement in computational capabilities now permits high-quality three-dimensional reconstruction of computed tomographic datasets. The three-dimensional reconstructions, particularly when achieved by volume rendering, permit visualization of cardiac anatomy equivalent to the dissection of heart specimens with the additional benefit of viewing in attitudinally appropriate orientation. Such dissection performed virtually has an added advantage of evaluating cardiac anatomy in vivo. Furthermore, the feasibility of reconstructing in any plane relevant to the cardiologist, radiologist or surgeon makes virtual dissection an ideal tool for education and clinical decision making. The ease and accuracy of cardiac anatomy using virtual dissection has prompted Professor Anderson to consider it as the gold standard for analysis of cardiac anatomy in living patients [1,2,3]. We are honored to contribute to this special issue of the journal, recognizing the monumental contributions that Professor Anderson has made to the field of cardiovascular medicine. In this review, we discuss the advantages and applications of volume-rendered images, specifically focusing on virtual dissection, in understanding patient-specific cardiac anatomy.

## 2. The Concept of Three-Dimensional Imaging Using the Volume-Rendering Method

Recent advances in visualization have permitted much needed three-dimensional reconstruction of datasets obtained from computed tomography and magnetic resonance imaging. Although commonly performed using commercial software packages, three-dimensional reconstruction can also be achieved using free open-source software [4,5,6,7,8]. These software are being increasingly used in clinical practice for diagnosis as well as to guide transcatheter and surgical intervention [9,10,11,12,13]. Some of these software have additional capability for creating and modifying the three-dimensional data to provide the appropriate file format for three-dimensional printing and various forms of extended reality [14,15]. Irrespective of the software and the visualization technique (virtual model on a monitor, printed model, or augmented/virtual reality), the general concept of three-dimensional imaging is the same in the regard that all three-dimensional visualization techniques rely on differential visualization of each iso- or anisotropic image voxel based on its attenuation density (computed tomography), signal intensity (magnetic resonance imaging), or intensity of echocardiographic signal (three-dimensional ultrasound). If a phasic dataset is obtained, four-dimensional images with time-dependent change in the cardiac anatomy can also be demonstrated (Supplementary Movies 1 and 2).

**Figure 1:**
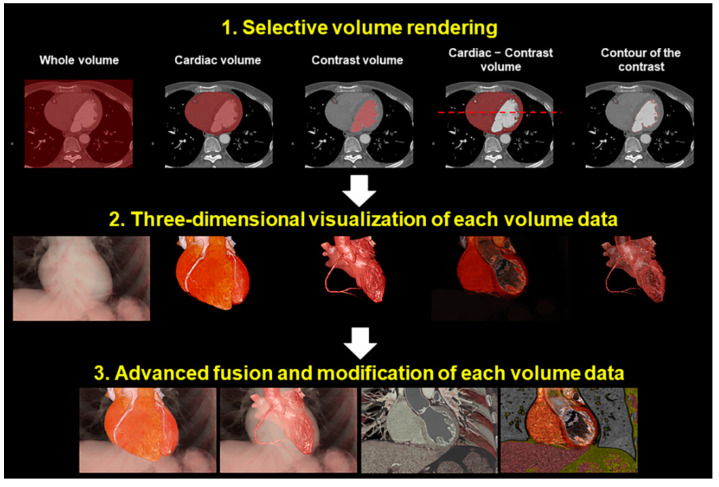
Concept of the image reconstruction. Each volume of interest can be reconstructed separately and can be merged at the reconstructors’ disposal.

**Figure 2:**
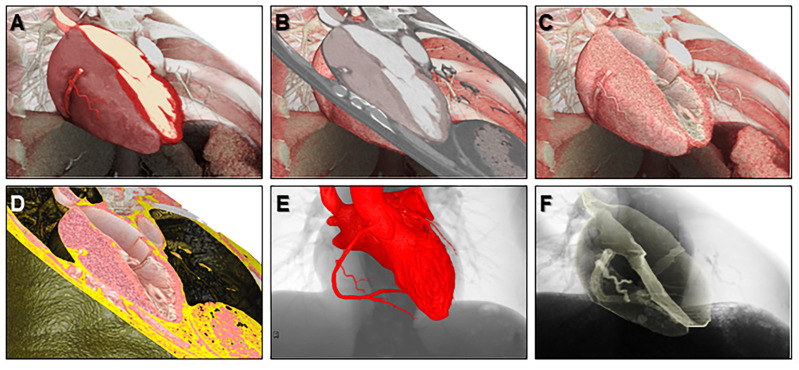
Representative various volume-rendered images reconstructed from a single dataset. We call these volume-rendering techniques 2.5-dimensional imaging (**A**,**B**), virtual dissection imaging (**C**,**D**), endocast imaging (**E**), and shell imaging (**F**).

Commonly, contrast enhancement is used for detailed cardiac evaluation to visualize the chambers, vessels, and/or coronary arteries. When the contrast agent is used, it is the enhanced chamber and/or the lumen of these vessels that are usually the focus of reconstruction (Figure 1 and Figure 2). We call this volume-rendering blood pool imaging as endocast images [1,16,17]. This is analogous to conventional die casting used for the dissected heart to create molds [18].

In this material, we mainly focused on the computed tomographic images as the spatial resolution of clinical datasets is commonly superior to that obtained from cardiac magnetic resonance imaging. However, three- or four-dimensional analysis is also feasible for datasets obtained from magnetic resonance imaging, which have advantages in its non-invasiveness, temporal resolution, tissue characterization, perfusion analysis, and flow analysis [19,20].

Virtual dissection is nothing but a modification of the volume-rendering technique wherein the focus is shifted to the walls of the cardiac chambers and vessels. While endocast images can be extremely useful, especially when visualizing the great vessels and their relationships to the other intrathoracic structures or relating to angiographic imaging, they become less informative when aiming to understand the detailed intracardiac anatomy and how this relates to the proximal great vessels. It is often the walls, septums, and valves of the chambers and/or vessels which are targeted for transcatheter or surgical intervention [17], information which can be demonstrated in exquisite detail by virtual dissection [21,22,23,24,25]. In this technique, the enhanced chamber is virtually removed from the original datasets by the process of manual thresholding. The non-enhanced walls, septums, and valves are then visualized to produce images similar to real dissection.

Depending on the interest of visualization, reconstruction using the volume-rendering method requires a combination of various concepts of imaging (Figure 1 and Figure 2).

## 3. The Advantage of the Virtual Dissection Compared to Conventional Real Dissection

**Figure 3:**
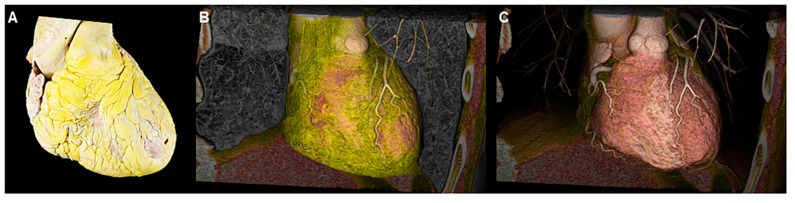
Real dissection (**A**) and virtual dissection images (**B**,**C**). When using virtual dissection images, attitudinal relationships with surrounding structures are maintained with easy modification on images to effectively show the structural anatomy of interest.

The main advantage of virtual dissection from computed tomographic datasets is that it can provide a high-resolution image of the blood-filled heart without removing it from the thorax (Figure 3). Unlike dissection of the heart specimens, the cut planes during virtual dissection are not fixed and are practically unlimited. Almost any view can be recreated for optimal visualization of the structures of interest as per the need. Each voxel retains its three-dimensional spatial coordinate, allowing the reconstructed image to show the details of living heart anatomy in attitudinally appropriated fashion [1]. In other words, the right, left, superior, inferior, anterior, and posterior directions are precisely demonstrated, allowing for accurate anatomical education and understanding. This is particularly important in providing relative orientation of cardiac structures with surrounding structures, including the esophagus, trachea and bronchi, lungs, diaphragm, nerves, vertebral column, and thorax itself [1]. Historically, the nomenclature of cardiac anatomy was based on describing the heart in the Valentine position, with the autopsied heart removed from the chest and placed on its apex. While much of this attitudinally incorrect nomenclature has been engrained in the cardiovascular fields, this creates misunderstanding and ambiguity when assessing various cardiovascular structures from common imaging modalities (i.e., angiography, cardiac magnetic resonance and computed tomography, etc.) or by intraoperative assessment with the heart in vivo [1,26,27]. Virtual dissection, on the other hand, by permitting constant guidance about relative orientation of the heart enables a similar attitudinally correct understanding of the in vivo cardiac anatomy by all members of the medical team. Virtual dissection also avoids the risk of being exposed by the potentially toxic fixatives used for specimen preservation, such as formalin. For these reasons, we believe the technique of virtual dissection from computed tomographic datasets has in many ways surpassed analysis of the autopsied heart as the gold standard for understanding detailed cardiac anatomy.

## 4. Educational Implication of the Three-Dimensional Living Heart Anatomy

It is well understood that “form begets function”. Therefore, it is not surprising that the foundation of medical education often begins with instruction in anatomy followed by (patho)physiology. While medical students are taught cardiac anatomy in their first years of medical education, supplemented by cadaveric dissection and inspection, the cardiology and cardiothoracic surgery trainees are often starved of such experiences. This relates to several factors, including the scarcity of preserved heart archives, competing educational requirements in a relatively limited time, and demanding clinical schedules [28,29,30]. So as to keep and improve the quality of anatomical knowledge, dissection-based teaching alone during medical school has to be complemented with newer teaching/learning methods, based on clinical imaging including computed tomography and magnetic resonance imaging [31,32,33]. Many cardiology trainees expand upon their knowledge in basic cardiac anatomy by interpretation of two-dimensional imaging modalities, primarily echocardiography. Assessing such a complex three-dimensional organ by two-dimensional means with a narrow field of view is fraught with error [34]. Education in cardiac anatomy using three-dimensional reconstructions has many advantages. There has been interest in using three-dimensional printed models for education albeit with the limitations of availability and costs. On most occasions, the assessment using printed models is also restricted to a singular plane of dissection [35]. Furthermore, similar to the heart specimens, freedom of moving the printed model extracted out of the chest cavity makes the assessment prone to errors. An unnatural tactile feeling of printed models also falls short of the real heart.

**Figure 4:**
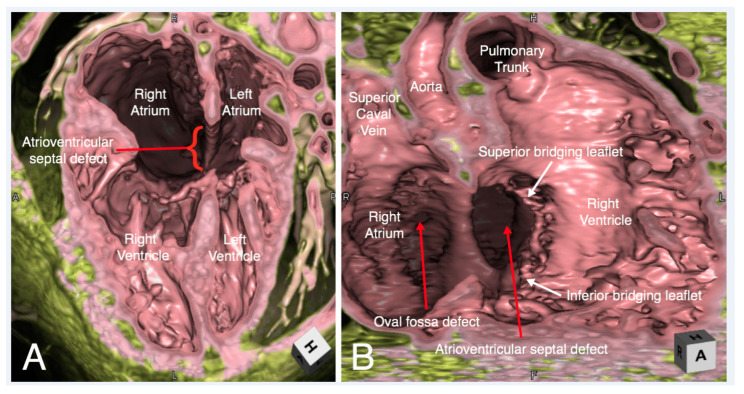
Virtual dissection of an atrioventricular septal defect in four-chamber (Panel **A**) and right anterior oblique (Panel **B**) planes. In the right anterior oblique plane (Panel **B**) the superior and inferior bridging leaflets are seen to have chordal attachments to the crest of the muscular interventricular septum, with small ventricular level shunting between the chordae. There is a large atrial component to the defect. There is additionally a small oval fossa defect.

**Figure 5:**
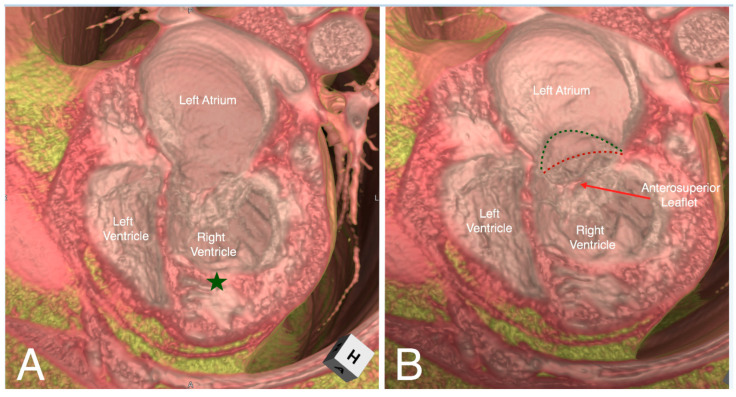
Virtual dissection of a patient with congenitally corrected transposition in a four-chamber plane demonstrating discordant atrioventricular connections with left-hand ventricular topology (Panel **A**). The moderator band (green star) and septal attachments of the left-sided atrioventricular valve identifies the left-sided ventricle as the morphological right ventricle. Tilting the dissected heart anteriorly and superiorly (Panel **B**), the hinge points of the septal and inferior tricuspid leaflets (red dashed line) are displaced inferiorly (the green dashed line represents its normal attachment points).

**Figure 6:**
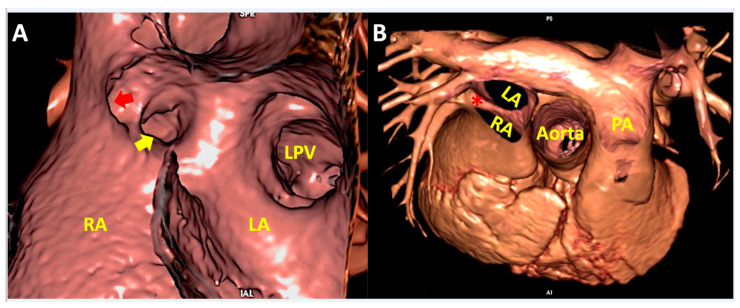
Virtual dissection of a superior sinus venosus interatrial communication in left anterior oblique projection (Panel **A**) and as viewed from the superior direction (Panel **B**). Panel **A** shows extraseptal location of the defect with anomalous connection of right superior pulmonary vein (red arrow) having retained connection to the left atrium. The right inferior pulmonary vein (yellow arrow) and left pulmonary veins connect normally to the left atrium. Panel **B** shows overriding of the superior caval vein on the atrial septum (*) when viewed from above. LA—left atrium; LPV—left pulmonary veins; PA—pulmonary artery; RA—right atrium.

**Figure 7:**
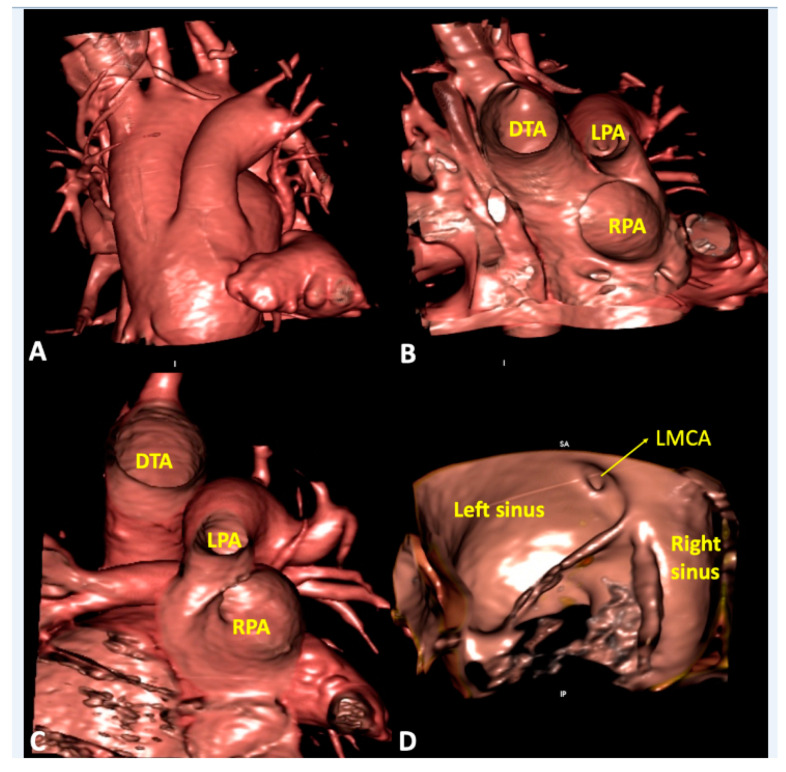
Virtual dissection of patients with common arterial trunk. (Panel **A** and **B**) from the same patient shows how it is difficult to clarify common versus separate origin of left and right pulmonary artery based on the external appearance (Panel **A**). The internal virtual dissection view (Panel **B**), on the other hand, clearly demonstrates separate origin of the pulmonary arteries. (Panel **C**) demonstrates crossed pulmonary arteries with the origin of the right pulmonary artery positioned to the left of the left pulmonary artery. (Panel **D**) shows juxtacommisural origin of left main coronary artery in a patient with bisinusate truncal valve. DTA—descending thoracic aorta; LMCA—left main coronary artery; LPA—left pulmonary artery; RPA—right pulmonary artery.

**Figure 8:**
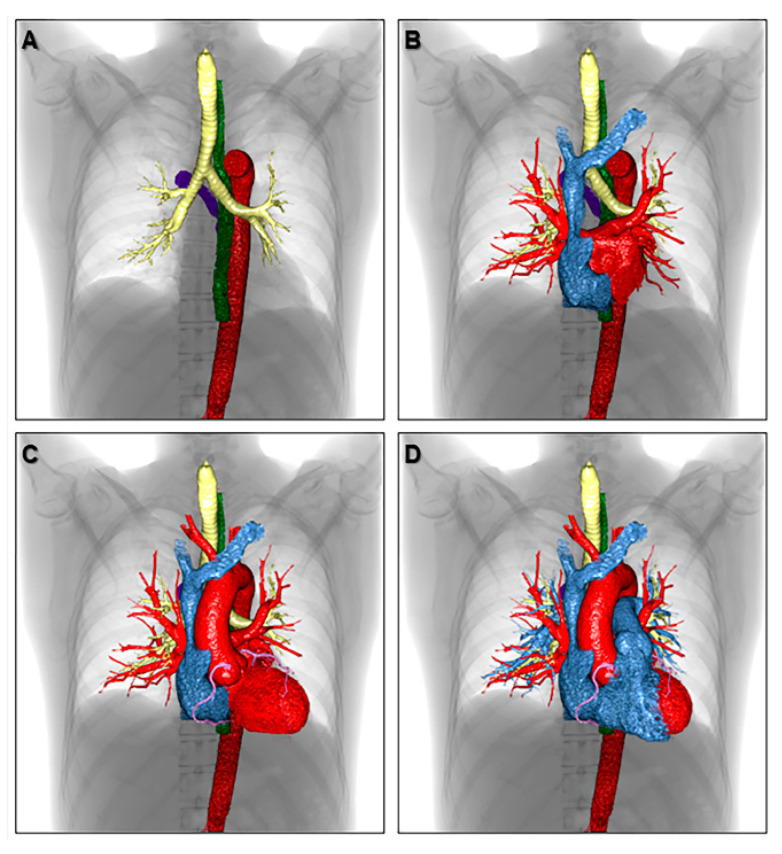
Volume-rendered images showing the three-dimensional relationship of each anatomical structure in the thorax. The azygos vein (purple), descending aorta (red), esophagus (green), trachea, and bronchi (yellow) are shown in panel (**A**). Both atria are added in panel (**B**). The left ventricle, aortic root, ascending aorta, aortic arch, and coronary artery are added in panel (**C**). The right ventricle, pulmonary root, pulmonary trunk, and pulmonary arteries are reconstructed in panel (**D**) to finalize the components of cardiac silhouette.

While three-dimensional imaging lacks the tactile advantage of the printed model of the heart, an excellent demonstration of cardiac anatomy in attitudinally appropriate orientation makes up for this deficiency to a large extent. Three-dimensional imaging has the potential to serve as the mainstay of imparting knowledge of cardiac anatomy in the absence of heart specimens [1]. Even at centers with a well-maintained archive of heart specimens, virtual dissection has a complementary role in educating students and health professionals. The ease of storing computed tomographic datasets in electronic format with the feasibility of creating endocast and/or virtual dissection images at any time practically immortalizes the anatomic details of any patient (Figure 4, Figure 5, Figure 6 and Figure 7) [35]. The electronic format also eliminates problems related to sharing of knowledge across institutions which is severely restricted with the use of heart specimens. Figure 8 shows the representative volume-rendered images usually used during routine educational lectures to show the three-dimensional relationship of each anatomical structure in the thorax.

## 5. Clinical Implication in Acquired Heart Disease with Representative Cases

**Figure 9:**
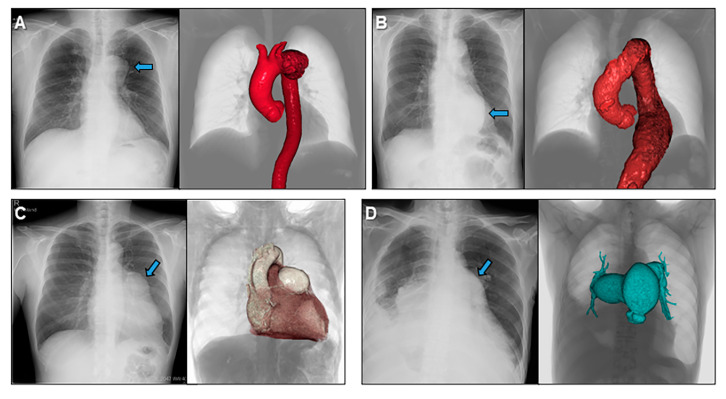
Volume-rendered images to understand abnormal chest radiographies showing a case with aneurysms of the aortic arch (**A**), descending aorta (**B**), left coronary artery (**C**), and pulmonary trunk and pulmonary arteries (**D**).

For educational and diagnostic purposes related to the heart, it is not always sufficient to understand only the cardiac anatomy. Often, it is more important to clarify relationships with the adjacent structures in the thoracic cavity [36,37,38]. Volume-rendered images are useful to relate to abnormal findings in chest radiography in individual cases (Figure 9).

**Figure 10:**
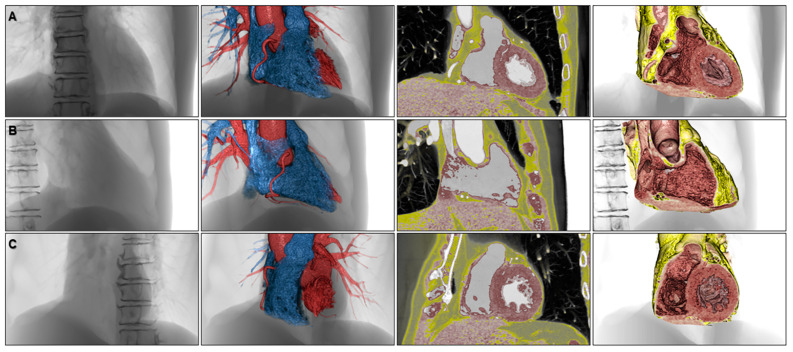
Volume-rendered images to appreciate fluoroscopic anatomy viewed from the frontal (**A**), right anterior oblique (**B**), and left anterior oblique (**C**) directions. The left panels, second left panels, second right panels, and right panels show fluoroscopy-like volume-rendered images, endocast images, 2.5-dimensional images, and virtual dissection images, respectively.

**Figure 11:**
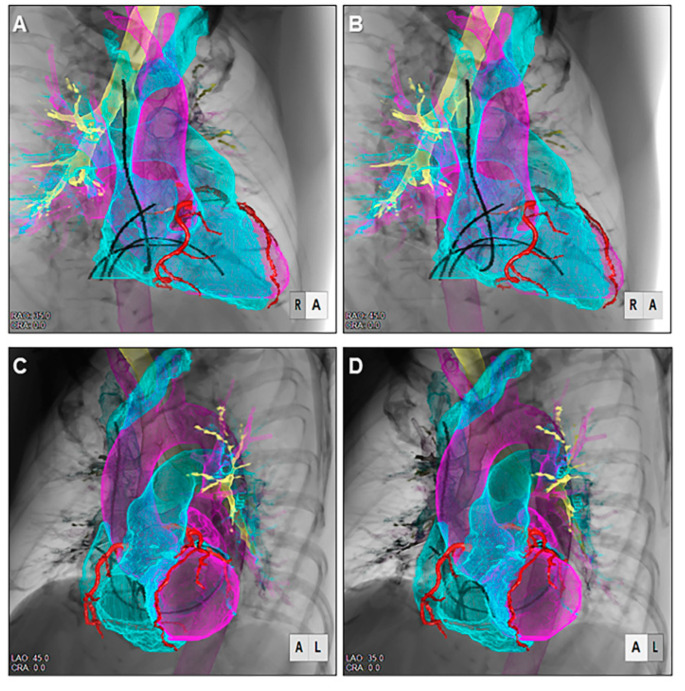
Stereoscopic displays (cross-eyed method) of virtual electrophysiologic catheters placed within the heart reconstructed using shell-imaging technique viewed from the right anterior oblique (**A**,**B**) and left anterior oblique (**C**,**D**) directions.

**Figure 12:**
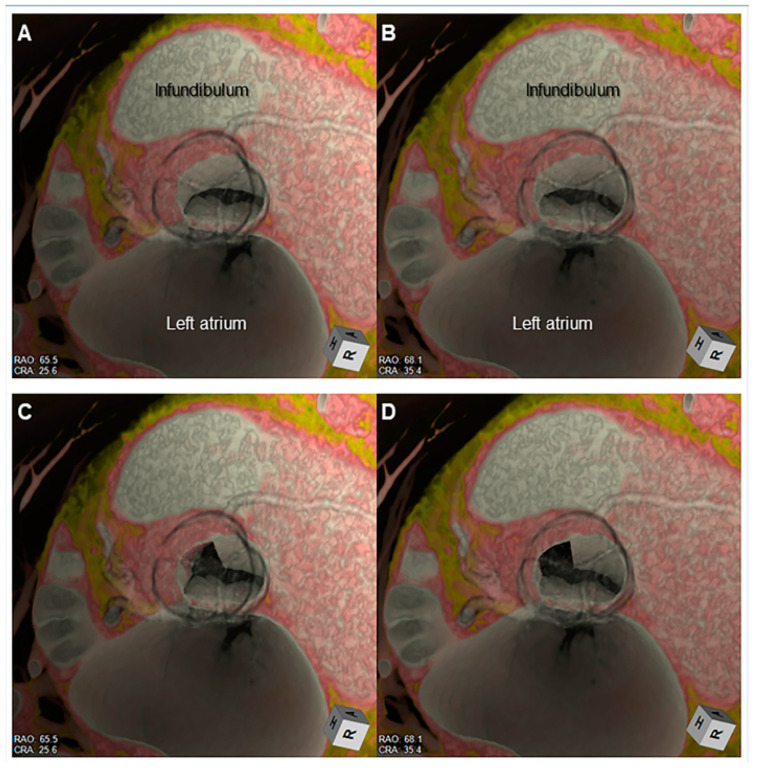
Stereoscopic displays (cross-eyed method) of virtual dissection images demonstrated from the surgeon’s view show presurgical virtual simulation of the Morrow’s septal myectomy. The hypertrophied septal myocardium located apical to the bottom of the right coronary aortic sinus (**A**,**B**) is virtually resected at its left half (**C**,**D**).

For more specific purposes, volume-rendered images, involving both endocast and virtual dissection images, can be also used to educate fluoroscopic anatomy to cardiac interventionists and electrophysiologists as their procedures are commonly performed under fluoroscopic guidance and with angiography (Figure 10). Virtual simulation of the invasive procedure, including electrophysiological study (Figure 11), biventricular pacing [39], radiofrequency catheter ablation, and His-bundle pacing are also feasible. Image demonstration from the surgeon’s view, as well as virtual simulation of the cardiac surgery in preoperative fashion is also feasible with stereoscopic presentation (Figure 12) [40].

## 6. Clinical Implication in Congenital Heart Disease with Representative Cases

Similar applications of three-dimensional reconstructions can be applied towards congenital heart disease. This includes not only in the evaluation of unrepaired congenital heart disease which may involve complex abnormal three-dimensional relationships of chambers and vessels, deficiencies in septation, or abnormalities of atrioventricular or semilunar valves but also in the evaluation of their repaired and palliated forms. A combination of both endocast and virtual dissection images is often beneficial to provide a comprehensive three-dimensional understanding. There is increasing application towards the adult congenital population, given the complexity of evaluating many of their repaired and palliated lesions as well as the common scenario of poor acoustic windows limiting complete echocardiographic evaluation [41].

**Figure 13:**
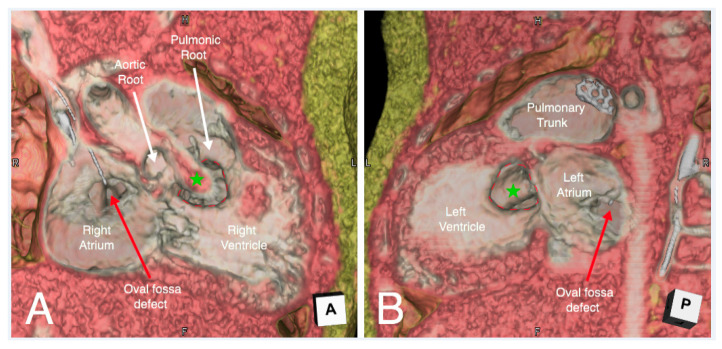
Virtual dissection of a patient with double outlet right ventricle with the interventricular communication (red dashed line) viewed from the right (Panel **A**) and left side (Panel **B**). The interventricular communication is doubly committed, bisected by the prominent outlet septum (green star). However, the potential pathway to the relatively larger pulmonary root is larger than that to the small aortic root with subaortic narrowing. There is a stent within the patent arterial duct seen entering the distal pulmonary trunk in this patient with a hypoplastic transverse aortic arch, coarctation of the aorta and ductal-dependent systemic arterial blood flow.

**Figure 14:**
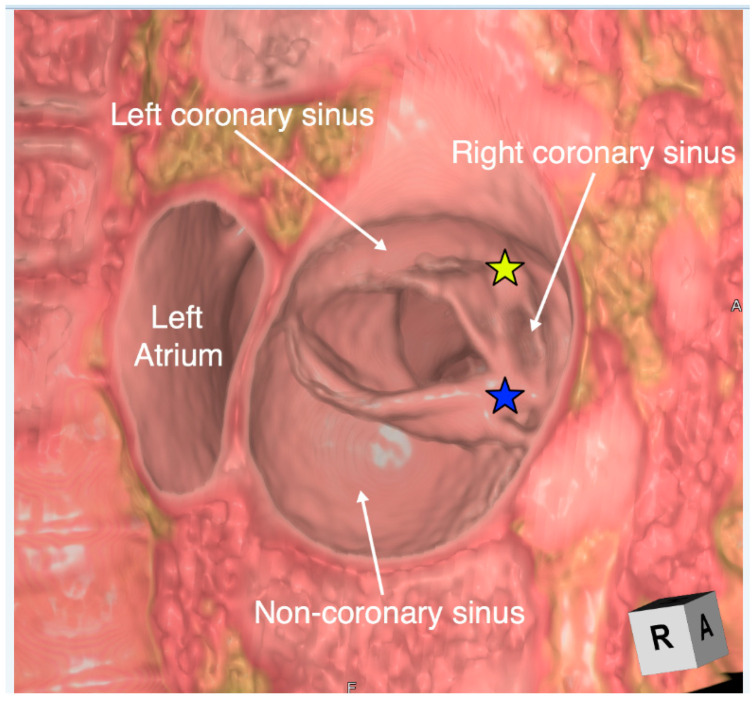
Virtual dissection of a unicuspid and unicommissural aortic valve viewed from the aorta. There is fusion with a raphe between both the right and left coronary leaflets (yellow star) and the right and non-coronary leaflets (blue star). A four-dimensional video is displayed in Supplemental Movie 2.

**Figure 15:**
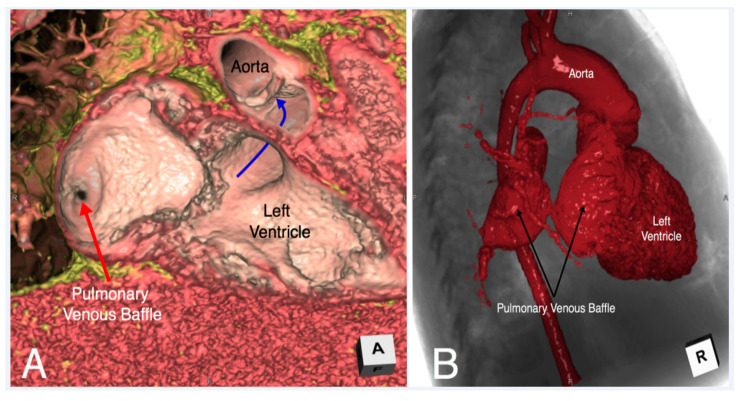
Virtual dissection (Panel **A**) and endocast reconstruction (Panel **B**) of a patient with congenitally corrected transposition status post double switch who developed severe obstruction of the pulmonary venous baffle. While the endocast image (Panel **B**) demonstrates the severe obstruction, it is the virtual dissection image (Panel **A**) which adds additional insight that the mechanism of obstruction is related to a membrane which has formed obstructing the pulmonary venous baffle.

**Figure 16:**
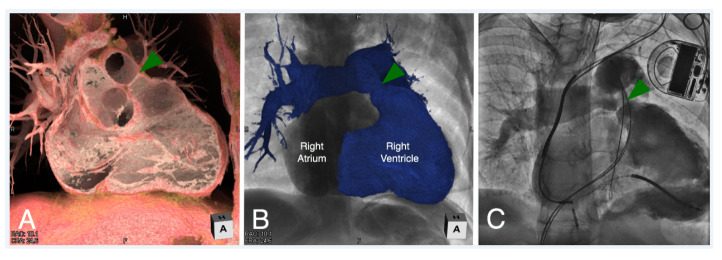
Virtual dissection (Panel **A**) and endocast reconstruction (Panel **B**) of a patient with repaired tetralogy of Fallot with moderate right ventricular outflow tract obstruction. Both images are viewed in a right anterior oblique plane. The virtual dissection image (Panel **A**) demonstrates a discrete ridge at the pulmonary sinutubular junction (green arrowhead). An angiogram was obtained in similar plane (Panel **C**) during cardiac catheterization confirming the anatomy, prior to placing a transcatheter pulmonary valve to relieve the obstruction.

In addition to cases demonstrated in Figure 4, Figure 5, Figure 6 and Figure 7, examples of three-dimensional images to guide interventional and surgical management in the congenital heart disease population are illustrated in the following:

- Relationship of the great arteries to an interventricular communication in the setting of double outlet right ventricle for surgical planning (Figure 13);
- Unicuspid aortic valve for surgical repair planning (Figure 14, Supplementary Movie 2);
- Obstruction in surgically constructed pulmonary venous baffles for surgical planning (Figure 15) [16];
- Right ventricular outflow tract obstruction in repaired tetralogy of Fallot to guide transcatheter pulmonary valve replacement (Figure 16).

## 7. Advanced Application of the Three-Dimensional Imaging

**Figure 17:**
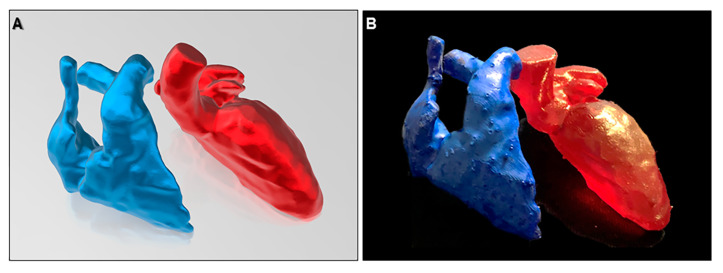
Virtual (**A**) and real (**B**) images of three-dimensional printing model of the right and left heart.

**Figure 18:**
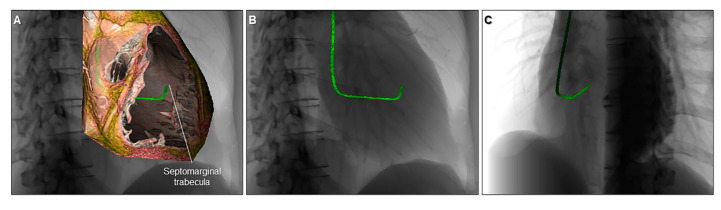
Virtual simulation of the catheter shape appropriate for right ventricular septal pacing visualized within the cardiac silhouette. The right ventricle is cut to show the septomarginal trabecula (**A**). The three-dimensional shape of the catheter is visualized in relation to the cardiac silhouette viewed from the right anterior oblique (**B**) and left anterior oblique (**C**) directions.

Three-dimensional imaging is currently used to create files for converting the image into three-dimensional printing [42] and virtual reality [33]. The printed model is useful for education as discussed above (Figure 17). Furthermore, it is also useful for surgeons for preoperational recognition of the complex anatomy, especially with the application of procedural simulation [12,43,44,45], as well as surgical training of the complicated procedures [46]. For this purpose, both endocast and virtual dissection images are used. Virtual reality supports the interactive sharing of the three-dimensional images during the simulated operation and also can guide the invasive procedures and operations, especially in such fields of orthopedics and neurosurgery [47,48]. As the heart is a highly moving structure, the application of virtual reality into the clinical cardiovascular fields can be challenging. However, many groups are exploring this application [15,45]. Three-dimensional images, when combined with some software which has extended application equivalent to computer aided design, allow customization of the device by virtually simulating its shape and course (Figure 18) [49].

There is no doubt three-dimensional printed models and the increasing use of augmented and virtual reality can be extremely useful for education, procedural simulation and planning [12,43,44,45]. However, in our experience, utilizing the endocast and virtual dissection images on a monitor is often the most optimal strategy in clinical practice to provide a detailed evaluation of three-dimensional cardiac anatomy to support transcatheter and surgical planning while balancing costs and time efficiency [4,11,12,16,50,51]. Three-dimensional images in this paper were reconstructed using commercially available software/workstation (Horos, Pixmeo, Geneva, Switzerland; Ziostation2; Ziosoft, Tokyo, Japan; 3D Builder, Microsoft, Redmond, WA, USA).

Furthermore, for the analysis of cadaveric hearts, three-dimensional imaging plays an important role to clarify three-dimensional arrangement of the myocardial mesh and conduction system using diffusion tensor magnetic resonance imaging and microcomputed tomography [52,53].

## 8. Limitations of Three-Dimensional Imaging and Importance of Basic Anatomical Knowledge and Multidisciplinary Relationship

When creating three-dimensional images based on the volume-rendering method, it should be always kept in mind that the quality of the final image depends on the quality of the raw data. In this regard, the collaboration of radiologists and radiological technologists is an important prerequisite to optimize image acquisition with appropriate radiation dose and contrast material [54]. While radiation exposure is a risk that must be weighed when considering alternative imaging options, with improving technology and the ability for minimal radiation exposure with modern scanners, the afforded spatial resolution has prompted increasing use of cardiac computed tomography [55].

**Figure 19:**
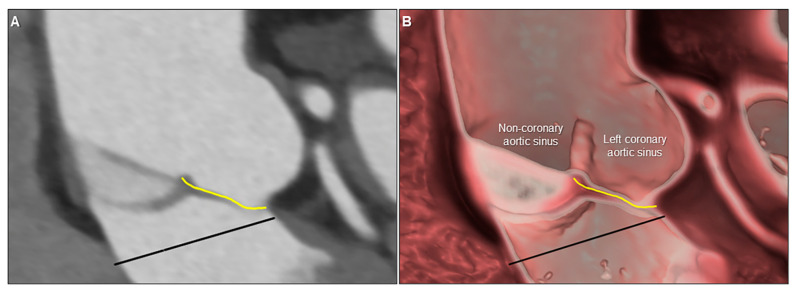
Two-dimensional multiplanar reconstruction (**A**) and three-dimensional virtual dissection (**B**) images showing the complicated anatomy of the aortic root. Yellow line and black line indicate geometric height and virtual basal ring plane, respectively. Measurement performed on the two-dimensional image can be projected on the three-dimensional image to secure the accuracy of the virtual dissection images.

The volume-rendered image might not precisely reflect the real anatomy. For example, the visualized anatomy can be enlarged or contracted from the real thing, depending on the software and window level/width applied. Thin or moving structures, such as the floor of the oval fossa (primary septum), membranous septum, and valvar leaflets are sometimes difficult to reconstruct, and can easily be mistaken for a defect. Some software can virtually create structures that are not present, such as vessels, as if it were a part of the real anatomy. Smoothing is commonly applied to some extent, which may distort the original information in the raw data. Therefore, measurements should not be performed on the volume-rendered image but should be measured using two-dimensional multiplanar reconstruction images. This can then be overlaid on the three-dimensional image for an improved understanding of these measurements related to the three-dimensional structure (Figure 19). Thus, to avoid creating images that can potentially mislead clinical judgement, it is paramount for the reconstructor to accurately interpret the anatomy based on the two-dimensional axial data [56]. It is the responsibility of the image creator to firmly visualize what can be seen in the raw data, and to acknowledge any structures which may be inaccurately demonstrated due to poor imaging quality. For this purpose, the most important basis for image reconstruction is the fundamental knowledge based on the real dissection-based anatomy, as we cannot visualize what we did not know [57]. Therefore, even if virtual dissection is considered the gold standard for anatomical analysis, as proposed by Professor Anderson [2], it is his achievements based on dissection-based cardiac anatomy that enables appropriate virtual dissection. Thus, virtual dissection can never completely replace the real dissection, but can surely provide complementary information relevant to educational and clinical management of patients with heart disease. Keeping good communication with radiologists, anatomists, clinical cardiologists, interventionalists, and surgeons is fundamental as these several departments are usually involved in the patient’s care. These means of continuous multidisciplinary communication not only positively impact those performing procedures, but with constructive feedback to the imager relating the intraoperative findings to the provided reconstructions, also improve the quality and accuracy of reconstructions.

## 9. Conclusions

The obtained three-dimensional data from each patient should be fully utilized to create images to show comprehensible clinical cardiac anatomy critical to the clinical diagnosis and treatment. In this regard, virtual dissection, a three-dimensional volume-rendering imaging technique, is emerging as the gold standard for understanding detailed cardiac anatomy in the living patient. This technique is useful for both medical education as well as a detailed clinical evaluation of the complex three-dimensional heart, whether demonstrating normal anatomy for educational purposes or evaluating the patient with acquired or congenital heart disease. Any reconstruction depends both on the source imaging data as well as the anatomical understanding of the reconstructor. It is a requirement for radiologists, anatomists, cardiologists, interventionalists and surgeons to keep abreast of the benefits and limitations of these techniques and to fully utilize the three-dimensional anatomical data obtained to optimize patient management and outcomes.

## Supplementary Materials

The following are available online at https://www.mdpi.com/2308-3425/7/3/30/s1, Supplementary Movie 1. Four-dimensional virtual dissection demonstrating the dynamic motion of the normal mitral valve throughout the cardiac cycle as viewed from the left atrium, Supplementary Movie 2. Four-dimensional virtual dissection demonstrating the dynamic motion of a unicuspid and unicommissural aortic valve. There is fusion with a raphe between both the right and left coronary leaflets and the right and non-coronary leaflets. The labeled image is displayed in Figure 14.
